# Can child pneumonia in low-resource settings be treated without antibiotics? A systematic review & meta-analysis

**DOI:** 10.7189/jogh.12.10007

**Published:** 2022-11-12

**Authors:** Patrick JB Walker, Chris Wilkes, Trevor Duke, Hamish R Graham, ARI Review group, Trevor Duke, Hamish Graham, Steve Graham, Amy Gray, Amanda Gwee, Claire von Mollendorf, Kim Mulholland, Fiona Russell, Maeve Hume-Nixon, Saniya Kazi, Priya Kevat, Eleanor Neal, Cattram Nguyen, Alicia Quach, Rita Reyburn, Kathleen Ryan, Patrick Walker, Chris Wilkes, Poh Chua, Yasir Bin Nisar, Jonathon Simon, Wilson Were

**Affiliations:** 1 Centre for International Child Health, Murdoch Children’s Research Institute, University of Melbourne, Royal Children’s Hospital, Parkville, Victoria, Australia; 2 Department of Paediatrics, University of Melbourne, Parkville, Victoria, Australia

## Abstract

**Background:**

WHO guidelines recommend the use of antibiotics for all cases of pneumonia in children, despite the majority being caused by viruses. We performed a systematic review and meta-analysis to determine which children aged 2-59 months with WHO-defined fast breathing pneumonia, if any, can be safely treated without antibiotics.

**Methods:**

We systematically searched medical databases for articles published in the last 20 years. We included both observational and interventional studies that compared antibiotics to no antibiotics in children aged 2-59 months diagnosed with fast breathing pneumonia in low- and middle-income countries (LMICs). We screened articles according to specified inclusion and exclusion criteria, and assessed for risk of bias using the Effective Public Health Practice Project (EPHPP) framework. Overall, we included 13 studies in this review. We performed a meta-analysis of four included studies comparing amoxicillin to placebo.

**Results:**

Most children with fast breathing pneumonia will have a good outcome, regardless of whether or not they are treated with antibiotics. Meta-analysis of four RCTs comparing amoxicillin to placebo for children with pneumonia showed higher risk of treatment failure in the placebo group (odds ratio OR 1.40, 95% confidence interval CI = 1.00-1.96). We did not identify any child pneumonia subgroups in whom antibiotics can be safely omitted. Limited data suggest that infants with clinically-diagnosed bronchiolitis are a particular low-mortality group who may be safely treated without antibiotics in some contexts.

**Conclusions:**

Children with WHO-defined fast breathing pneumonia in LMICs should continue to be treated with antibiotics. Future studies should seek to identify which children stand to benefit most from antibiotic therapy, and identify those in whom antibiotics may not be required, and in which circumstances.

Pneumonia is the leading single cause of death in children under five, killing an estimated 740 000 children in 2019 [1]. WHO guidance recommends antibiotics for all children meeting WHO’s broad clinical definition for pneumonia (cough and fast or difficult breathing) [2,3]. WHO guidance does not identify any patient groups in whom antibiotics may not be needed despite the majority of pneumonia episodes being caused by viruses [2,3]. This approach is designed to ensure children with bacterial pneumonia at risk of death do not go without antibiotic therapy, but has meant that many children with viral infections are being treated with antibiotics. This treatment paradigm its in tension with antimicrobial stewardship efforts, intended to optimise the use of antimicrobials, improve patient outcomes, prevent antimicrobial resistance, and save health care costs [4-8]. In the era of widespread uptake of vaccination against *Streptococcus pneumoniae* and *Haemophilus influenzae* this has become even more important, as the proportion of cases caused by bacterial pathogens continues to falll [9].

Differentiation between bacterial and viral pneumonia is challenging and clinical signs alone are often unreliable [10]. A number of studies have examined the utility of biomarkers including white cell count (WCC), C-reactive protein (CRP), and procalcitonin (PCT), in conjunction with clinical symptoms and examination findings, in determining likely aetiology. These studies have shown somewhat inconsistent findings, generally indicating that biomarkers may have a role in identifying children with bacterial rather than viral infection but are insufficiently sensitive to “rule out” bacterial infection [11-13]. The usefulness of these biomarkers to health care workers (and patients) using WHO guidelines in low- and middle-income countries (LMICs) is limited further by low availability and high relative cost [11-13].

For infants with clinically-diagnosed bronchiolitis, WHO hospital guidelines acknowledge that, in the absence of signs of severe illness or danger signs, these children are unlikely to benefit from antibiotics [3]. However, the WHO pneumonia case definition is broad, including all children with “cough and fast or difficult breathing”. This means that many children in LMICs with uncomplicated bronchiolitis are likely to be treated with antibiotics, as they fulfil WHO criteria for pneumonia despite having a viral infection which is unlikely to require antibiotics [14].

We conducted a systematic review and meta-analysis to examine current evidence on the effectiveness of antibiotics in children with WHO-defined fast breathing pneumonia in LMICs and determine which children, if any, can be safely managed without antibiotics, and in which contexts.

## METHODS

### Types of studies

We included studies that: 1) were published in English in the year 2000 or later; 2) included children with WHO-defined fast breathing pneumonia; 3) included children who were treated with antibiotics and children who were treated without antibiotics; and 4) were wholly or partially undertaken in LMICs. We included both interventional and observational studies. Full inclusion and exclusion criteria are detailed in **Table 1**.

**Table 1:** Inclusion and exclusion criteria

Inclusion criteria	Exclusion criteria
**Observational or interventional study or meta-analysis involving original data or analysis**	Does not provide original data or analysis (eg, review articles, editorials)
**Published in the year 2000 or later**	Conducted in a neonatal unit/neonatal ICU, or focuses only on neonates below 40 weeks’ gestation
**Published in English**	All included children were treated with antibiotics, or all children were treated without antibiotics
**Includes children aged between 28 d and 14 y of age**	Definition of pneumonia used is not clearly described or inconsistent
**Includes children whose presenting problem is pneumonia as defined by WHO, which may include other acute respiratory infections such as bronchiolitis**	
**Includes children with WHO-defined pneumonia who were treated without antibiotics**	
**Wholly or partially undertaken in LMICs, as defined by World Bank**	

WHO – World Health Organization, LMICs – low- and middle-income countries, ICU – intensive care unit

### Search strategy

We developed the protocol for this systematic review and meta-analysis in accordance with PRISMA reporting guidelines [15,16]. We conducted a systematic search of medical databases Medline, Embase, and PubMed for all relevant articles. We mapped search terms to medical subject headings where possible, using Boolean operators to combine searches into our final systematic search query. We used synonyms of ‘pneumonia’, ‘antibiotics’, and ‘child’ to target our search strategy, with oversight from an experienced Health Service Librarian to ensure all relevant papers were identified. The specific search terms used for our Medline search are included in Appendix S1 IN the **Online Supplementary Document**. We also searched reference lists of all included references for eligible studies.

### Assessment of study eligibility

Two reviewers, PW and CW, independently screened the titles and abstracts of all returned studies. We obtained full-text for studies that were screened in by either reviewer, and the same two reviewers independently assessed them for inclusion. We resolved disagreements by discussion and, where appropriate, review by a third reviewer, HG. None of the reviewers were blind to the journal titles, study authors, or affiliated institutions.

### Data management, extraction and synthesis

We used a standardised data extraction form to extract data relevant to our review. Two reviewers, PW and CW, independently extracted data from each eligible study and entered data into an Excel spreadsheet (Microsoft, Redmond, US). We resolved disagreements by discussion, and contacted study authors where appropriate to resolve any uncertainties. Types of data extracted are listed in Table S1 in the **Online Supplementary Document**.

### Meta-analysis

We conducted a meta-analysis of four RCTs [17-20] comparing oral amoxicillin to placebo in children diagnosed with fast breathing pneumonia. These studies had similar patient populations, similar methodologies, and similar primary outcomes (treatment failure at day 3 or 4). We used Stata 17.0 (StataCorp, College Station, TX, USA) to perform the meta-analysis, using raw data from each of the four studies imported into Stata using Microsoft Excel (Microsoft Corporation, NM, USA). We used a random effects model, and reported outcomes both as odds ratios (using exponentiated effect sizes) We set the confidence level at 95%.

### Assessment of study quality and risk of bias

We assessed the quality and risk of bias of all included studies by using the Effective Public Health Practice Project (EPHPP) Quality Assessment Tool [21,22]. Using this tool, two reviewers, PW and CW, independently rated studies as strong, moderate or weak with respect to selection bias, study design, confounders, blinding, data collection method, withdrawals and dropouts, and a global rating. Where disagreements occurred, a third reviewer, HG, carried out a final assessment. Risk of bias of each study is included in Table S2 in the **Online Supplementary Document**.

## RESULTS

**Figure 1:**
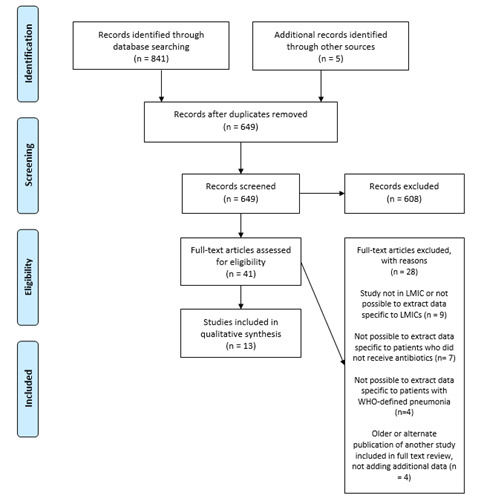
PRISMA flow chart for inclusion of studies. LMIC – low- or middle-income country, WHO – World Health Organization.

Our database search returned 841 records, and review of references returned an additional five papers. After removing duplicates, 649 papers were eligible for screening. We excluded 608 papers based on title and abstract screening, and a further 28 papers based on full-text review. Overall, we included 13 papers in our review (**Figure 1**).

We included four randomised-controlled trials (RCTs) which compared antibiotic therapy with placebo in children with WHO-defined fast breathing pneumonia (previously known as non-severe pneumonia) [17-20], and five RCTs which compared antibiotic therapy to no antibiotic therapy in children with clinically diagnosed bronchiolitis [23-27]. We also included two RCT sub-analyses [28,29], one cohort study [30], and one systematic review [31], all of which looked at the utility of antibiotic therapy in children with fast breathing pneumonia. Detailed characteristics of included studies can be found in **Table 2**.

**Table 2:** Detailed characteristics of included studies

Study authors	Year	Study type	Loca-tion	No of partic-ipants	Participant characteristics	Exclusion criteria	Number excluded	Use of pulse oxim-etry	Intervention and control	Primary outcome	Follow-up period & safety net	Study quality
*Randomised controlled trials comparing antibiotics to no antibiotics in children with WHO-defined fast breathing pneumonia*												
**Awasthi et al [** 17 **]**	2008	RCT	India (SEARO, LMIC)	1671	• 2-59mo • Outpatient, hospital • WHO-defined fast breathing pneumonia with wheeze • Tachypnoea despite bronchodilator therapy • PCV/Hib vaccine status not reported	• Severe or very severe pneumonia • Congenital heart disease • Chronic systemic disorders • Other condition requiring antibiotics • Hospitalisation in past 2 weeks • Antibiotic use in past 48 h • Measles in past month • Radiological pneumonia • Penicillin allergy	1813 (1605 had normal RR following salbutamol; 206 had radiological pneumonia; 2 refused consent)	All	**I:** Oral amoxicillin, 31-54mg/kg/d in 3 divided doses for 3 d; and oral salbutamol, 2.5 or 5ml TDS for age 2-11mo and 12-59mo respectively for 3 d **C:** Oral placebo matched in appearance and taste to oral amoxicillin, and oral salbutamol as for intervention group	Proportion of treatment failure on day 4 (development of severe or very severe pneumonia at any stage; SpO2 < 90% at any stage; axillary temperature >101F at any stage; persistence of fast breathing pneumonia on day 4; or presence of wheeze on day 4)	14 d; Home visits within 24h for patients who failed to report	Strong
**Ginsburg et al [** 18 **]**	2019	RCT	Malawi (AFRO, LIC)	1126	• 2-59mo • Outpatient, hospital • WHO-defined fast breathing pneumonia • Tachypnoea despite bronchodilator therapy • 55.0% received PCV • 55.1% received pentavalent Hib vaccine	• Severe respiratory distress or chest indrawing • WHO danger signs • SpO2 < 90% • Stridor when calm • HIV-1 positive or exposed • Severe acute malnutrition • Tuberculosis (or suspected) • Severe anaemia • Severe malaria • Hospitalisation in past 2 weeks • Antibiotic use in past 48 h • Different conditions requiring antibiotics • Penicillin allergy • Participated in different clinic study in previous 12 weeks	217 (166 did not meet inclusion criteria; 61 declined to participate)	All	**I:** Oral amoxicillin 250mg dispersible tablets in 2 divided doses, 500mg/d for 2-11mo, 1000mg/d for 12-35mo, 1500mg/d for 36-59mo for 3 d **C:** Oral placebo dispersible tablets, identical in appearance, smell, taste, dispersion activity and packaging to amoxicillin used in study, also given twice daily for 3 d	Proportion of treatment failure on day 4 (development of WHO general danger signs or severe respiratory distress, at any stage; SpO2 < 90 at any stage; RR at any stage ≥10 higher than at enrolment; not able to be discharged from hospital, if admitted for any reason)	14 d; Phone calls days 1-3, in-person clinical review days 2-4 & day 14	Strong
**Hazir et al [** 19 **]**	2011	RCT	Pakistan (EMRO, LMIC)	873	• 2-59mo • Outpatient, hospital • WHO-defined fast breathing pneumonia • PCV/Hib vaccine status not reported	• Underlying chronic illness • History of ≥3 episodes of wheeze or acute bronchial asthma • Antibiotic use in past 48 h	1203 (1087 did not meet inclusion criteria; 26 refused to participate; 90 for ‘other reasons’)	Not reported	**I:** Oral amoxicillin 15mg/kg 3 times per day for 3 d **C:** Placebo tablet with similar colour and consistency in the same volume, masked with regard to taste and smell	Proportion of treatment failure by day 3 (development of WHO general danger signs or lower chest indrawing by day 3)	14 d; Clinic review days 3, 5 and 14; 2 home reviews if failed to report	Strong
**Jehan et al [** 20 **]**	2020	RCT	Pakistan (EMRO, LMIC)	4002	• 2-59mo • Primary health centres • WHO-defined fast breathing pneumonia • Tachypnoea despite bronchodilator therapy • 61.5% received PCV and/or Hib vaccine	• Chest indrawing or any WHO danger sign • Known tuberculosis • Asthma • Pedal oedema • Other severe illness requiring antibiotics • Hospitalisation in past 2 weeks • Antibiotic use in past 48 h	3885 (2169 did not meet inclusion criteria; 483 declined to participate; 1233 ‘had other reasons’	All	**I:** Oral amoxicillin 500mg BD if 4-10kg, 1000mg BD if 10-14kg, 1500mg BD if 14-20 kg, for 3 d **C:** Matched volume of placebo, also taken BD for 3 d	Proportion of treatment failure after day 3 (death, development of WHO danger signs or chest indrawing, admission to hospital, or development of a new-onset infection or serious adverse event requiring the trial regimen to be changed)	14 d; Clinic review days 5, 8, 12 & 14; home visits if failed to report; 24-h hotline available for all participants	Strong
*Sub-analyses of trials comparing antibiotics to no antibiotics in children with WHO-defined fast breathing pneumonia*												
**Keitel et al [** 28 **]**	2019	RCT subgroup analysis	Tanzania (AFRO, LMIC)	711	• 2-59mo • Outpatient depts. of 3 district hospitals and 6 urban health centres • Fever and cough without signs of severe illness (those with WHO-defined fast breathing pneumonia only included in this review) • 96% received PCV • 97% received Hib vaccine	• Weight <2.5kg • Main complaint of injury or acute poisoning • Previous medical care for presenting illness	1537 (1391 did not meet inclusion criteria; 146 not recruited)	All	**I (ePOCT):** Children with cough and tachypnoea or chest indrawing unresponsive to bronchodilator therapy *and* a CRP>80mg/L, and all children with SpO2 < 90%, received oral amoxicillin 80-100mg/kg/d. Those without both of the above criteria (clinical signs of pneumonia and CRP>80) did not receive amoxicillin **C (ALMANACH):** Children were treated with antibiotics as per existing IMCI guidelines (using existing ALMANACH decision-making tool)	Proportion of treatment failure by day 7	30 d; Clinic review day 3, phone review day 14 and 30; caregivers asked to re-present if concerned about deterioration	Mod-erate
**Nkwopara et al [** 29 **]**	2019	RCT sub-analysis	Malawi (AFRO, LIC)	1126	As per Ginsburg et al: • 2-59mo • Outpatient, hospital • WHO-defined fast breathing pneumonia • Tachypnoea despite bronchodilator therapy • 55.0% received PCV • 55.1% received pentavalent Hib vaccine	As per Ginsburg et al: • Severe respiratory distress or chest indrawing • WHO danger signs • SpO2 < 90% • Stridor when calm • HIV-1 positive or exposed • Severe acute malnutrition • Tuberculosis (or suspected) • Severe anaemia • Severe malaria • Hospitalisation in past 2 weeks • Antibiotic use in past 48 h • Different conditions requiring antibiotics • Penicillin allergy • Participated in different clinic study in previous 12 weeks	As per Ginsburg et al: 217 (166 did not meet inclusion criteria; 61 declined to participate)	All	As per Ginsburg et al: **I:** Oral amoxicillin 250mg dispersible tablets in 2 divided doses, 500mg/d for 2-11mo, 1000mg/d for 12-35mo, 1500mg/d for 36-59mo for 3 d **C:** Oral placebo dispersible tablets, identical in appearance, smell, taste, dispersion activity and packaging to amoxicillin used in study, also given twice daily for 3 d	Proportion of serious adverse events (events that resulted in death, were life-threatening, required admission to hospital or prolongation of hospital admission, resulted in persistent or significant disability or incapacity, or jeopardised the health of the participating child or required medical or surgical intervention)	14 d; Phone calls days 1-3, in-person clinical review days 2-4 & day 14	Strong
**King et al [** 30 **]**	2016	Exploratory sub-analysis of an observational prospective cohort study	Malawi (AFRO, LIC)	847	• 2-59mo • Community-level primary care clinics • WHO-defined fast breathing pneumonia • Wheeze not assessed nor recorded	• Lower chest wall indrawing • WHO danger signs • Absence of tachypnoea • Incomplete treatment (ie, commenced antibiotics but did not complete prescribed course)	775 (487 not eligible (too old or without fast breathing); 197 lost to follow-up; 91 non-adherent or TF not determined)	All	**I:** Co-trimoxazole and/or lumefantrine-artemether **C:** No antibiotic or antimalarial treatment	Rate of non-recovery (persistence of fast-breathing, fever, lower chest-indrawing, any danger sign, change of antibiotic, hospital admission, or death) at day 5	14 d; Home visits day 5 & 14, referral to CHW if required for assessment and on-referral	Weak
*Randomised controlled trials comparing antibiotics to no antibiotics in children with bronchiolitis*												
**Kabir et al [** 23 **]**	2009	RCT	Bangladesh (SEARO, LMIC)	295	• 0-24mo • Inpatient, hospital • Clinical diagnosis of bronchiolitis, including chest indrawing and wheeze on auscultation • PCV/Hib vaccine status not reported	• Atopic conditions • Congenital heart disease • Possible immunodeficiency • Chronic lung problem • Associated infection • Previous antibiotic use	146 (114 did not meet inclusion criteria, 32 lost to follow-up)	All	**I_1_:** IV ampicillin, 50mg/kg/dose given 6-hourly **I_2_:** Oral erythromycin, 10mg/kg/dose given 6-hourly **C:** No antibiotics **Note**: all patients received 6-hourly nebulised salbutamol along with supportive therapy	Hospital length of stay	7 d; 8-hourly medical review, no review post-discharge	Mod-erate
**Mazumder et al [** 24 **]**	2009	RCT	Bangladesh (SEARO, LMIC)	126	• 1-24mo • Outpatient and inpatient, hospital • Clinical diagnosis of bronchiolitis, including chest indrawing and wheeze on auscultation • PCV/Hib vaccine status not reported	• Atopic conditions • Congenital heart disease • High fever >102°F • Toxic appearance	Not specified	All	**I_1_:** IV ampicillin, 100-200mg/kg/dose given 6-hourly **!_2_:** Oral erythromycin, 30-50mg/kg/d given 6-hourly **C:** No antibiotics **Note**: all patients received 6-hourly nebulised salbutamol along with supportive therapy	Clinical improvement or discharge from hospital (if hospitalised)	7 d; 3 reviews per day if inpatient, 2 reviews per day if outpatient, referral availability not reported	Weak
**Pinto et al [** 25 **]**	2012	RCT	Brazil (PAHO, UMIC)	184	• 0-12mo • Emergency depts of 2 tertiary hospitals • Clinical diagnosis of bronchiolitis with less than 72h of LRTI symptoms • PCV/Hib vaccine status not reported	• Chronic cardiopulmonary disorder • Congenital or acquired immunodeficiency • Neuromuscular disease • Prematurity or other neonatal complications • Macrolides contraindicated • Chlamydia species or pertussis respiratory infection	4 (reason not specified)	All	**I:** Oral azithromycin 10mg/kg daily for 7 d **C:** Equivalent volume of placebo, also given daily for 7 d	Hospital length of stay	Until discharge from hospital; Frequency of review not reported	Mod-erate
**Rasul et al [** 26 **]**	2008	RCT	Bangladesh (SEARO, LMIC)	60	• 0-24mo • Inpatient, hospital • Clinical diagnosis of bronchiolitis, including chest indrawing • PCV/Hib vaccine status not reported	• History of atopy • Congenital heart disease • Known immunodeficiency	Not specified	All	**I_1_:** IV amoxicillin, dose and duration not specified **I_2_:** Oral erythromycin, dose and duration not specified **C:** No antibiotics	Proportion of clinical improvement vs deterioration as deemed by investigators	Until discharge from hospital; Frequency of review not reported	Weak
**Tahan et al [** 27 **]**	2007	RCT	Turkey (EURO, UMIC)	21	• 0-7mo • Inpatient, hospital • Clinical diagnosis of bronchiolitis; first episode of wheeze requiring hospitalisation • PCV/Hib vaccine status not reported	• Cardiac disease • Cystic fibrosis • Chronic neonatal lung disease associated with prematurity • Steroids within past 24 h • Bronchodilators within 4 h of presentation	Not specified	All	**I:** Oral clarithromycin 15mg/kg daily for 3 weeks **C:** Matched placebo, also given daily for 3 weeks	Hospital length of stay	Until discharge from hospital; Frequency of review not reported	Mod-erate
*Non-randomised studies included in this review*												
**Lassi et al [** 31 **]**	2014	Syste-matic review	Global	0 papers	• 2-59mo • Setting not specified • RCTs of children with WHO-defined fast breathing pneumonia and wheeze	• Severe or very severe pneumonia • Any chronic illness • Any other condition requiring antibiotics • Measles within past month • Hospitalisation in past 2 weeks • Antibiotic use in past 48 h	N/A	N/A	**I:** Any antibiotic therapy **C:** No medical treatment or placebo	Proportion of clinical cure (temperature and RR decreased to normal) vs treatment failure (development of WHO danger signs, chest indrawing, or RR elevated for age at completion of treatment)	N/A	N/A

WHO – World Health Organization, RCT – randomised controlled trial, SEARO – Regional Office for South-East Asia, LMIC – lower-middle income country, PCV – pneumococcal conjugate vaccine, Hib – *Haemophilus influenzae* type B, RR – respiratory rate, I – intervention, C – control, TDS – three times per day, AFRO – Regional Office for Africa, LIC – low-income country, HIV – human immunodeficiency virus, SAM – severe acute malnutrition, TB – tuberculosis, EMRO – Regional Office for the Eastern Mediterranean, BD – twice per day, PAHO – Regional Office for the Americas, UMIC – upper-middle income country, LRTI – lower respiratory tract infection, EURO – Regional Office for Europe, TF – treatment failure, CHW – community health-worker

### Antibiotics vs placebo in children with WHO-defined fast breathing pneumonia

All of the four RCTs comparing antibiotics to placebo in children with pneumonia took place in low- or lower-middle income countries (two in Pakistan, one in India, and one in Malawi). Three took place in hospital outpatient departments and one was conducted in primary health centres. Each trial included children with fast breathing pneumonia as defined by WHO (cough or difficult breathing with chest indrawing or fast breathing for age) [32], and excluded children who responded to inhaled bronchodilators and those with WHO general danger signs (inability to drink or breastfeed, vomiting everything, convulsions, lethargy, or unconsciousness). All four trials used oral amoxicillin in the intervention arm and evaluated “treatment failure” at day 3 or 4 as the main clinical outcome and relapse at day 14 as a secondary outcome. Treatment failure definitions varied slightly between studies, typically including death, hypoxaemia, WHO emergency or severe pneumonia signs, chest wall indrawing, or admission to hospital. Notably, Awasthi et al included presence of wheeze despite treatment in their definition of treatment failure [17]. Primary outcomes reported were treatment failure at day 3 [19] or day 4 [17,18.20].

**Figure 2:**
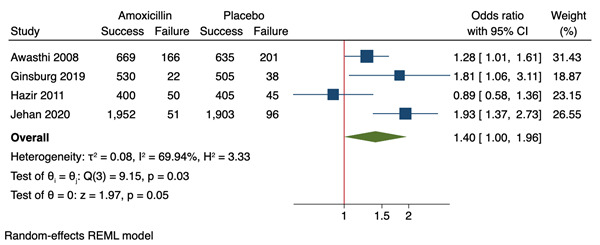
Meta-analysis forest plot describing risk of treatment failure by day 3 or 4 in children with WHO-defined fast breathing pneumonia. 95% CI – 95% confidence interval.

Three trials, which together included 6799 children, found that treatment with placebo was associated with a significantly higher rate of treatment failure by day 4 (OR range 1.28-1.92, 95% CI range = 1.01-2.70) [17,18,20]. The remaining trial, which included 873 children, found no difference (OR 0.88, 95% CI = 0.57-1.39) (**Table 3**) [19]. Treatment failure rates were low in both antibiotic and placebo groups with moderate variability between studies: median 7.6% (range 2.5-19.9) vs 8.5% (range 4.8 to 24.1). Meta-analysis showed a significantly higher rate of treatment failure in children treated with placebo than those treated with amoxicillin (OR 1.40, 95% CI = 1.00-1.96; RD 2%, 95% CI = 1%-3%) (**Figure 2**). Overall, 3551/3840 (92.5%) of children who received amoxicillin and 3448/3828 (90.1%) of children who received placebo had good day 4 outcome and, 3448 (90.1%) had a good outcome and, of those cured on day 4, 3.9% (136/3524) of the amoxicillin group and 3.4% (116/3408) of the placebo group had relapsed at day 14.

**Table 3:** Key results from randomised controlled trials comparing antibiotics to no antibiotics in children with WHO-defined fast breathing pneumonia

Study	Number of participants	Proportion treatment failure at day 3 or 4	Comparison	Relapse at day 14 in those cured by day 4	Comparison	Mortality
**Awasthi et al, 2008 [** 17 **]**	1671	Amoxicillin: 166/835 (19.9%) Placebo: 201/836 (24.1%)	Adjusted OR = 1.28 (95% CI = 1.01-1.62) RD = 4.2	Amoxicillin: 41/669 (6.1%) Placebo: 44/635 (6.9%)	RD = 0.8% (-1.9-3.5). *P* = 0.6	Amoxicillin: 0 Placebo: 0
**Ginsburg et al, 2019 [** 18 **]**	1126	Amoxicillin: 22/552* (4.0%) Placebo: 38/543* (7.0%)	Adjusted RR = 1.78 (1.07-2.97) RD = 3	Amoxicillin: 34/530* (6.4%) Placebo: 26/505* (5.1%)	Adjusted RR = 0.80 (0.49-1.32)	Amoxicillin: 0 Placebo: 0
**Hazir et al, 2011 [** 19 **]**	900	Amoxicillin: 50/450 (11.1%) Placebo: 45/450 (10.0%)	OR^†^ = 0.88 (0.57-1.39). RD = 1.1, *P* = 0.59	Amoxicillin: 3/373 (0.8%)* Placebo: 5/364 (1.4%)	OR = 0.58 (0.11-2.81) *P* = 0.46	Amoxicillin: 0 Placebo: 0
**Jehan et al, 2020 [** 20 **]**	4002	Amoxicillin: 51/2003 (2.5%) Placebo: 96/1999 (4.8%)	OR^†^ = 1.92 (1.35-2.70). RD = 2.3 (0.9-3.6)	Amoxicillin: 58/1952 (3.0%) Placebo: 41/1904 (2.2%)	RD = -0.8 (-2.0, 0.3)	Amoxicillin: 1/2003 (0.05%) Placebo: 1/1999 (0.05%)

OR – odds ratio, 95% CI – 95% confidence interval, RD – risk difference

Intention-to-treat results used unless otherwise stated; statistically significant results underlined

95% confidence intervals reported in parentheses following odds ratios unless otherwise stated

*Per-protocol figures (ITT not reported).

^†^Figures from original paper adjusted (inverted) to enable comparison of studies.

Mortality was exceptionally low and similar between antibiotic and placebo groups (1/3840 vs 1/3828). A secondary analysis of Ginsburg et al’s trial [29] found that few participants reported any severe adverse effects (SAEs) in either the placebo (9.6%) or amoxycillin (7.8%) group and only 5/102 (4.9%) SAEs were possibly related to amoxicillin [29]. Awasthi et al reported 30 hospitalisations due to adverse effects with no deaths [17], and Jehan et al reported low rates of adverse effects (3.0% and 2.1% and 3.0% of participants in the amoxicillin and placebo groups, respectively) [20].

None of the included trials identified any population subgroups in whom placebo was as effective as amoxicillin. Jehan et al found increased rates of treatment failure for those without antibiotics were still evident after defining subgroups according to age, presence or absence of fever, and presence or absence of wheeze [20]. Similarly, Ginsburg et al found that none of age, respiratory rate, malnutrition, malaria, or pneumococcal conjugate or pentavalent vaccine status affected rates of treatment failure [18]. However, a number of factors were associated with an increased risk of treatment failure across both antibiotic and placebo groups: presence of wheeze, tachypnoea, fever, vomiting, and diarrhoea [19,20].

All the included trials specified strict exclusion criteria, including clinical signs of severity (eg, WHO general danger signs) as well as comorbid conditions and malnutrition. All trials included regular clinical review, typically 2-3 times during the first week and 1-2 times in the second week, and most provided parents with a phone number to call if concerned. All trials used pulse oximetry routinely (except Hazir et al., [19] which did not report pulse oximetry use), excluding children who had hypoxaemia at baseline and using hypoxaemia as a key marker of treatment failure.

A single secondary analysis of an RCT evaluated the effect of using C-reactive protein (CRP) in addition to pulse oximetry as part of a treatment algorithm to guide antibiotic use. This study, conducted in hospital outpatient departments and urban health centres in Tanzania, reported a lower rate of day 7 treatment failure compared to usual care (OR 0.60, 95% CI = 0.37-0.98) and significantly reduced antibiotic use [28]. A sub-analysis of a cohort study of rural Malawian children with fast-breathing pneumonia seen by community health workers compared children who were treated with co-trimoxazole with those who were not. On univariate analysis co-trimoxazole was associated with lower levels of treatment failure on day 5 (OR 0.34, 95% CI = 0.16-0.74) however, this was not significant on multivariate analysis [30]. One Cochrane review conducted in 2014 looked at the effectiveness of antibiotics in children with fast breathing pneumonia and wheeze but did not identify any studies which met the authors’ inclusion criteria [31].

Mortality was low in all studies included in this review, with four children out of 8383 dying (overall case fatality rate = 0.05%). Jehan et al reported one death in each group [20], and Keitel et al reported two deaths in the control (standard of care) group [28]. The remaining studies all reported zero deaths.

### Antibiotics vs no antibiotics in children with clinically-diagnosed bronchiolitis

We included five RCTs looking at the use of antibiotics in children aged less than 7-24 months with bronchiolitis. These studies all took place in hospital settings in middle-income countries (Bangladesh x3, Brazil, Turkey), and mostly included admitted patients. Definitions used of bronchiolitis varied, however all involved a clinical diagnosis by treating clinicians based on symptoms such as coryza, cough, difficulty in breathing, chest indrawing, and in one study [27] RSV positivity on nasopharyngeal aspiration. While these studies restricted inclusion to children with bronchiolitis rather than pneumonia, the vast majority would have met the broad WHO diagnostic criteria for pneumonia (or severe pneumonia). Antibiotic regimens varied between the studies, with three trials comparing both intravenous and oral antibiotics to no antibiotics, and two trials comparing oral macrolide antibiotics with placebo. Aside from Kabir et al’s trial, which excluded 146 out of 441 recruited participants [23], exclusion rates were low. Reasons for exclusions were not well reported.

Results of these studies were mixed (**Table 4**). Kabir et al [23] found that treatment without antibiotics was associated with a reduced hospital length of stay compared to both IV and oral antibiotics (3.67 ± 1.45 days vs 4.29 ± 1.89 and 4.44 ± 1.93 days respectively, *P* < 0.001), whereas Tahan et al [27] found that oral clarithromycin was associated with a reduced length of stay compared with placebo (51 vs 88 hours, IQR 48-68 and 71-100h respectively). The two other trials that reported length of stay found no significant difference between antibiotics and no antibiotics. Only Tahan et al. found a significant difference in clinical improvement rates, finding better outcomes with use of clarithromycin (*P* < 0.05), though this study only included 21 children. Only two trials reported mortality, both reporting zero deaths [24,26]. All participants of all trials underwent pulse oximetry, and were not discharged from hospital or outpatient follow-up until specified discharge criteria were met. Frequency of review, safety net and escalation procedure for deterioration post-discharge was not reported in any trial.

**Table 4:** Key results from randomised controlled trials comparing antibiotics to no antibiotics in children with bronchiolitis

Study	Number of parti-cipants	Hospital length of stay	Compa-rison	Clinical improvement	Compa-rison	Mortality
**Kabir et al, 2009 [** 23 **]**	295	IV ampicillin: 4.29 ± 1.89 d Oral erythromycin: 4.44 ± 1.93 d Control: 3.67 ± 1.45 d*	*P* <0.001	IV ampicillin: 90%-99% (various symptoms & signs) Oral erythromycin: 88%-98% (various symptoms & signs) Control: 94%-99% (various symptoms & signs)	*P* = 0.07-0.96	Not reported Not reported Not reported Not reported
**Mazum-der et al, 2009 [** 24 **]**	126	IV ampicillin: Not reported Oral erythromycin: Not reported Control: Not reported	N/A	IV ampicillin: 29/29 (100%) Oral erythromycin: 32/32 (100%) Control: 43/43 (100%)	Time to recovery: *P* = 0.66. Improve-ment in feeding: *P* = 0.23. Resolution of tachyp-noea: *P* = 0.05	0 0 0
**Pinto et al, 2012 [** 25 **]**	184	Oral azithromycin: 5.0 d (2.0-6.0) Placebo: 5.0 d (3.0-7.0)^†^	*P* = 0.29	Oral azithromycin: Not reportet Placebo: Not reported	N/A	Not reported Not reported
**Rasul et al, 2008 [** 26 **]**	60	IV amoxicillin: 6.7 ± 1.1 d Oral erythromycin: 6.3 ± 1.5 d Control: 6.2 ± 1.4 d*	*P* > 0.1	IV amoxicillin: 22/23 (95.5%) Oral erythromycin: 21/22 (95.5%) Control: 14/15 (92.9%)	*P* > 0.5	0 0 0
**Tahan et al, 2007 [** 27 **]**	21	Oral clarithromycin: 51 h (48-68) Placebo: 88 h (71-100)^†^	*P* <0.05	Oral clarithromycin: 11/12 (91.7%) Placebo: 5/9 (55.6%)^‡^	*P* <0.05	Not reported Not reported

Statistically significant results underlined; *P* values are reported as they were reported in individual studies. Ranges are reported only where more specific *P* values were not available.

*Mean ± standard deviation.

^†^Median (interquartile range).

^‡^Defined as no re-admission to hospital post-discharge.

## DISCUSSION

This review included 13 papers which assessed the efficacy of antibiotics in children with WHO defined fast breathing pneumonia and bronchiolitis. We found that treatment failure rates were low regardless of antibiotic use. However, in three of four high-quality RCTs directly comparing amoxicillin to placebo, amoxicillin was associated with lower rates of treatment failure. Our meta-analysis showed that children treated with amoxicillin have 40% higher odds of treatment success than those treated with placebo (OR 1.40, 95% CI = 1.00-1.96). In children with bronchiolitis, antibiotics do not appear to have a significant benefit. Mortality rates, where reported, were very low regardless of antibiotic use.

Our results indicate that the majority of children with WHO-defined fast breathing pneumonia will have a good outcome regardless of treatment with or without antibiotics, though the rate of treatment failure is likely to be lower with use of amoxicillin. Included studies did not identify population subgroups in whom antibiotics are less likely to be beneficial. Specifically, wheeze, malaria, malnutrition, age, and pneumococcal conjugate and pentavalent vaccination status were not associated with any difference in response to antibiotics. As such, current evidence does not support the use of these factors to discriminate between patients who should or should not receive antibiotics. Further research in this area is needed to reliably determine which patients are most likely to benefit from antibiotics, and in which children antibiotics may be safely withheld.

We included one study [28] which examined a CRP-informed strategy (ePOCT) to guide antibiotic use, in which non-hypoxaemic children with cough and tachypnoea and a CRP over 80mg/L were given amoxicillin, and those without an elevated CRP were not. This trial found a significantly lower rate of treatment failure on day 7 among children treated using the CRP-informed strategy (RR 0.60, 95% CI = 0.37-0.98), and significantly lower antibiotic use. A 2016 study of patients with acute respiratory infection in Vietnam which included both children and adults similarly showed reduced antibiotic use in the group in which treatment was guided by CRP, without significant difference in treatment failure or death [33]. Sub-analysis of the PERCH trial, published in 2017, similarly found that elevated CRP was associated with bacterial infection, highlighting it as a potentially useful biomarker to identify children with bacterial infection [11]. More recent data from Malaysia involving 300 children with very severe pneumonia found that male sex, crepitations and elevated CRP were associated with higher risk of bacterial infection [34]. These data suggest that strategies which involve CRP and other inflammatory biomarkers may be helpful in determining which children stand to benefit most from antibiotics, particularly when used in conjunction with clinical signs. However, more research is needed in this area, and particularly in children with fast breathing pneumonia, including data on cost implications. Given their imperfect sensitivity and specificity, care must also be taken to ensure that these biomarkers are used as an adjunct to clinical signs rather than used as a standalone diagnostic tool, to ensure serious bacterial infections in children with low markers are not missed [35].

In children with clinically-diagnosed bronchiolitis, current research from LMICs included in this review supports current WHO guidance that antibiotics are not necessary in the absence of signs of pneumonia. There is, however, considerable overlap in the presentation of bronchiolitis and pneumonia, with many children satisfying criteria for both conditions. In these cases, where the treating clinician cannot be certain that the correct diagnosis is bronchiolitis, it may be safest to treat with antibiotics.

Evidence suggests that viral-bacterial co-infection is common in children with both pneumonia and bronchiolitis, meaning antibiotic use may be useful even in children with suspected or known viral infection [36,37]. This complicates the question of when antibiotics may be safely withheld, as despite the fact that viruses cause the majority of cases, bacterial co-infection is challenging to confidently rule out [2]. Given this uncertainty, treating these children with antibiotics remains the safest option in many settings.

While the studies included in this review that examined antibiotic use in children with WHO-defined fast breathing pneumonia are of high quality and have a low risk of bias, care needs to be taken when applying these results to a real-world context. Included studies had strict inclusion criteria, generally excluding children with chronic illnesses, including malnutrition, HIV, congenital heart disease, and chronic respiratory illnesses (**Table 2**). These children are likely to experience higher rates of treatment failure than other children, and the studies included in this review are unlikely to be helpful in determining when to use antibiotics in these children. Exclusion rates were generally high, with some studies excluding more than half of children screened. In the four RCTs which included children with WHO-defined pneumonia, more than 7000 children were excluded, mostly due to not meeting strict inclusion criteria (**Table 2**). This potentially limits clinicians’ ability to confidently translate the studies’ results to their clinical practice. Included studies also reported universal pulse oximetry use, allowing children with hypoxaemia to be reliably identified. Pulse oximetry is superior to clinical signs in detecting hypoxaemia and is an essential clinical tool in caring for sick children [38]. Previous research indicates many children with pneumonia in LMICs do not have access to pulse oximetry [38], and as such many cases of hypoxaemia, particularly if mild, are likely to be unrecorded. Hypoxaemia is a major risk factor for death among children with pneumonia [39,40], and absence of universal pulse oximetry may therefore limit the generalisability of the studies included in this review. Further, additional factors such as housing, proximity and access to health care facilities, maternal literacy, and financial position may also influence children’s ability to seek medical attention should they deteriorate. The studies included in this review which examined the efficacy of antibiotics in fast breathing pneumonia generally reported escalation and referral procedures and an ability to carry out home visits where participants failed to report as planned. This degree of safety net is unlikely to be present in many settings in LMICs, meaning deterioration may not be able to be detected as easily. This should be taken into consideration when deciding whether antibiotics can be safely withheld, to avoid harm in children who cannot be reviewed at home or referred easily should they deteriorate.

## CONCLUSIONS

Most children with mild acute lower respiratory tract infection characterised only by fast breathing, but without chest-indrawing or danger signs – those WHO defines as having fast breathing pneumonia – will experience a good outcome regardless of whether they are treated with antibiotics or not. However, current evidence suggests that use of oral amoxicillin is associated with a lower rate of treatment failure, and as such should be used when treating these children in LMICs. Children in whom a diagnosis of bronchiolitis can confidently be made, and who do not satisfy WHO criteria for pneumonia, can be safely treated without antibiotics. Further research is needed to identify subgroups of children with pneumonia in whom antibiotics may be safely withheld, and in which circumstances.

## Additional material


Online Supplementary Document
