# Supplementary material for: Can child pneumonia in low-resource settings be treated without antibiotics? A systematic review & meta-analysis
Source: J Glob Health. 2022 Nov 12;12:10007. doi: 10.7189/jogh.12.10007 (PMC9653171; doi:10.7189/jogh.12.10007)
Supplement: Online Supplementary Document [file jogh-12-10007-s001.pdf]

Online Supplementary Document

Appendix S1: Search terms used in Medline literature search
Database: Ovid MEDLINE(R) ALL <1946 to August 24, 2020>
Search Strategy:
----
1 exp *Pneumonia/ (83279)
2 ((respiratory adj3 (infection* or distress or failure or disease* or illness*)) or pneumonia or pneumonias or lung-inflammation* or lobitis or nonspecific-inflammatory-lung-disease* or peripneumonia or pleuropneumonia or pleuropneumonitis or pneumonic-lung* or pneumonic-pleurisy or pneumonic-pleuritis or pneumonitides or pneumonitis or pulmonal-inflammation* or pulmonary-inflammation* or pulmonic-inflammation* or bronchiolitis).tw,kf. (296596)
3 *Pneumococcal Infections/ (10122)
4 exp *Bronchiolitis/ (6586)
5 1 or 2 or 3 or 4 (333892)
6 exp *Anti-Bacterial Agents/ or *anti-infective agents/ or exp *Penicillins/ (486373)
7 Penicillin*.tw,kf. (60690)
8 (antibiotic or antibiotics or ((antibacterial* or anti-bacterial* or bacteriocid* or antimicrobial* or anti-microbial* or antimycrobial* or anti-mycrobial* or antiinfective or anti-infective) adj1 agent*)).tw,kf. (375245)
9 exp *amoxicillin/ (5367)
10 (a-gram or abdimox or acilina or acimox or actimoxi or adbiotin or agerpen or alfamox or alfoxil or almodan or almorsan or alphamox or amagesen-solutab or ameclina or amitron or amo-flamisan or amo-flamsian or amocillin or amoclen or amodex or amoflux or amohexal or amolin or amonex or amopen or amophar-ge or amosine or amoval or amoxa or amoxal or amoxapen or amoxaren or amoxcil or amoxcillin or amoxcin or amoxi-basan or amoxicilina or amoxicilline or amoxiclin or amoxicot or amoxidal or amoxidin or amoxidrops or amoxihexal or amoxil or amoxillin or amoxina or amoxipen or amoxipenil or amoxisol or amoxivan or amoxivet or amoxy or amoxycillin or amoxycilline or ampliron or apo-amoxi or ardine or aroxin or azillin or bacihexal or bactamox or bactox-ge or beamoxy or betamox or bimox or bintamox or biomox or biotamoxal or bioxidona or bioxyllin or bristamox or brl-2333 or brl2333 or broadmetz or cabermox or cilamox or clamox or clamoxyl or clearamox or clonamox or coamoxi
n or damoxicil or dispermox or doxamil or draximox or edamox or efpinex or erphamoxy or eupen or farconcil or fisamox or flemoxin or flemoxine-ge or fluamoxina or foxolin or fullcilina or gexcil or gimalxina or glamox or glassatan or gomcillin or grinsul or grunamox or hamoxillin or hiconcil or hidramox or hipen or hosboral or ibamox or ibiamox or ikamoxil or imacillin or imaxilin or inamox or infectomycin or intermox or isimoxin or izoltil or julphamox or jutamox or kamoxin or ladoxillin or lamoxy or larocilin or larocin or larotid or macromox or magnimox or maxamox or maxcil or medimox or meixil or metifarma or mopen or morgenxil or

moxacin or moxaline or moxarin or moxilen or moxilin or moximar or moxitab or moxtid or moxylin or moxypen or moxyvit or neogram or novabritine or novamox or novamoxin or novenzymin or novoxil or nuvosyl or optium or oramox or ospamox or pamocil or pamoxicillin or pamoxin or panvilon or pasetocin or penamox or penbiosyn or pentyloxycillin or pharmoxyl or piramox or polymox or pondnoxcill or rancil or ranmoxy or ranoxil or ranoxyl or robamox or romoxil or ronemox or saltermox or sawacillin or sawamezin or servamox or shamoxil or sia-mox or sigamopen or sil-a-mox or silamox or simoxil or sintopen or solamocta or solpenox or sumox or superpeni or teramoxyl or tolodina or tormoxin or triafamox or triamoxil or trifamox or trimox or uro clamoxyl or uroclamoxyl or utimox or vastamox or velamox or vistrep or widecillin or winpen or wymox or xiltrop or zamocillin or zamox or zamoxil or zerrsox or zimox).tw,kf. (15939)

11 *ampicillin/ (5722)

12 (acillin or aldribid or aletmicina or alfasilin or alpha-aminobenzylpenicillin or alphacin or ambiopi or amblocin or amblosin or amcill or amcillin or amficot or amfipen or aminobenzylpenicillin or amipenix or amoxi or amoxine or ampcillin or ampecu or ampen or ampenolet or ampensaar or ampexin or ampibex or ampiblan or ampicher or ampicil or ampicilin or ampicilina or ampiciline or ampicillin or ampicilline or ampicin or ampicyn or ampidar or ampifen or ampiflex or ampiger or ampilag or ampillin or ampimedin or ampipen or ampitenk or ampivral or ampkid or amplacilina or amplibin or ampliblan or amplital or amplivacil or ampolin or ampycin or amsapen or anglopen or anhypen or apo-ampi or austrapen or ay-6108 or ay6108 or bayer-5427 or binotal or biocil or bremcillin or bridopen or britapen or brl-1341 or brl1341 or c-10575 or c10575 or camicil or cetampin or cimexillin or citicil or clovillin or copharcilin or dhacillin or diferin or doktacillin or doltirol or domicill
in or dotirol or duacillin or dumopen or eracillin or eurocin or excillin or extrapen or fontapen or gramcil or h-ambiotico or helvecillin or herpen or hi-63 or hi63 or hostes or ibimycin or ikacillin or intramed or iwacillin or jenampin or julphapen or ks-r1 or marticil or mecil-n or neosensitabs or nuvapen or omnipen or p-50 or pamecil or panacta or pen-an or penbristol or penbritin or penbritin-s or penicline or penodil or penstabil or pentrex or pentrexil or pentrexyl or petercillin or pfizerpen-a or picylin or polycillin or polyflex or polypen or pricillin or primapen or princillin or principen or radiocillina or redicillin or rimacillin or roscillin or semicillin or servicillin or shacillin or sintelin or standacillin or standcillin or synpenin or synthocilin or synthocillin or tolimal or totacillin or totapen or trafarbiot or tricil or trifalicina or trihypen or trilaxin or ukapen or usampi or vacillin or viccillin or vidopen or virucil or vitapen).tw,kf. (25550)

13 *sulfamethoxazole/ or *trimethoprim, sulfamethoxazole drug combination/ or *Trimethoprim/ or *beta-Lactams/ (10137)

14 (Abactrim or abactrin or alfatrim or apo-sulfatrim or bactar or bactipront or bactoreduct-forte or bactramin or bactrim or bactrimel or bethaprim or biseptol or bispetol or chemotrim or co-trimoxazole or comox or comoxol or cotrim or cotrimoxazol-forte or cotrimoxazole or cotrimstada-forte or deprim or drylin-forte or duobact or duobiocin or duratrimet or eltrianyl or escoprim or espectrin or eusaprim or fectrim or groprim or helveprim or imexim or infectrim or kepinol or lagaprim or

lagatrim or linaris or microtrim or neoprim or nopil or oecotrim or omsat or oribact or oriprim or pharmaprim or potesept or resprim or resprin or ro-6-2580-11 or ro-6-258011 or ro-62580 or scanprin or septra or septran or septrim or septrin or septrine or sigaprim or sinersol or soltrim or sulfamethoprim or sulfamethoxazole-and-trimethoprim or sulfamethoxazole-plus-trimethoprim or sulfamethoxazole-trimethoprim or sulfaprim or sulfatrim or sulmeprim or sulprim or sumetrolim or sumetrolin o
r supracombin or thiocuran or tms-forte or trib or trigonyl or trimeth-sulfa or trimethoprim-plus-sulfamethoxazole or trimethoprim-sulfamethoxazole or trimethoprimsulfamethoxazole or trimetoprim-sulfa or trimetoprimsulfamethoxazole or trimezol or trimforte or trimosulfa or trimoxazole or trimoxol or uro-ts-d or uroplus-ds or uroplus-ss).tw,kf. (15929)
15 *penicillin g/ (4174)
16 (Angicillin or Bacinol or benzopenicillin or benzyl-penicillin* or benzylpenicillin or benzylpenicillinic-acid or benzylpenicilloyl or bupenna or cilligram or cilloral or cilopen or compocillin-g or confets or conspen or dis-pen or dramcillin-100 or drop-cillin or dropcillin or gelacillin or gynocillin or iricillin or jacillin or jenets or k-cillin or keracillin or liquacillin or liquapen or megapen or nebutab or novocillin or novocilline or pen-eff or penaire or penalev or penasoid or pendex or peneucin or penichrysin or penicillan or penicilline or penicilline-g or penioral or penisem or pentab or penzal or perolety or pharmacillin or phenylmethylpenicillin or pradupen or purapen-g or readcillin or retarpen or sol-tabs or solupen or sopen or specillin-g or stanpen-500 or sugracillin or til-pen or unicilina or vagicillin).tw,kf. (2954)
17 exp *Gentamicins/ (10461)
18 (Adelanin or alcomicin or apigent or apogen or apoten or azupel or bactiderm or biogaracin or bristagen or cidomycin or danigen or dermogen or dianfarma or dispagent or duragentam or duragentamicin or epigent or frieso-gent or g-mycin or garabiotic or garalone or garamicin or garamicina or garamycin or garbilocin or gencin or gendril or genoptic or genrex or gensumycin or genta or gentabiotic or gentabiox or gentac or gentacidin or gentacin or gentacor or gentacyl or gentafair or gentagram or gentak or gental or gentaline or gentalline or gentalol or gentalyn or gentamax or gentame or gentamedical or gentamen or gentamerck or gentamicin or gentamicins or gentamina or gentamycin or gentamycine or gentamyl or gentamytrex or gentaplus or gentarad or gentasil or gentasol or gentasone or gentasporin or gentatrim or genticin or genticina or genticyn or gentiderm or gentimycin or gentocin or gentogram or gentomycin or genum or geomycine or gevramycin or grammicin or hexamycin
or jenamicin or konigen or lacromycin or lisagent or martigenta or megental or migenta or miragenta or miramycin or nichogencin or nsc-82261 or nsc82261 or obogen or ocu-mycin or ocugenta or oftagen or ophtagram or opthagen or opti-genta or optigen or ottogenta or pyogenta or refobacin or ribomicin or rigaminol or rocy-gen or rovixida or rupegen or sagestam or sch-9724 or sch9724 or sedanazin or servigenta or skinfect or sulmycin or tangyn or terramycin or u-gencin or versigen or yectamicina).tw,kf. (28852)

19 *cephalosporins/ or *cefotaxime/ or *ceftriaxone/ (18330)
20 (Acantex or axone or benaxona or biotrakson or biotriax or bioxon or broadced or brospec or cef-3 or cefaflox or cefalogen or cefatriaxone or cefaxona or cefaxone or cefin or cefotal or cefotriaxon or cefotriaxone or cefriex or ceftrex or ceftrian or ceftriaxone or ceftrilem or cefxon or cephin or cephtriaxone or cerixon or cikedrix or citeral or ecotrixon or elpicef or eurocef or exempla or ferfacef or forgram or glicocef or gomcephin or grifotriaxona or incephin or keftriaxon or keptrix or lopratin or lyceft or medoxonum or megion or mesporin or monocef or nakaxone or novosef or oframax or pantrixon or retrokor or rinxofay or ro-13-9904 or ro139904 or rocefalin-roche or rocefin or rocephalin or rocephin or rocephine or rocidar or rowecef or roxcef or roxon or samixon or sintrex or socef or sunflow or tacex or torocef-1 or trexofin or triaken or triax or triaxone or tricefin or tricephin or trijec or xtenda or zefaxone or zefone-250).tw,kf. (11110)
21 exp *Erythromycin/ or exp *Macrolides/ (60732)
22 (macrolide* or macrocyclic-lactone-antibiotic-agent* or macrotetrolide*).tw,kf. (16506)
23 (Aruzilina or atizor or azadose or azasite or azatril or azenil or azibiot or azimin or azithral or azithromycin or azitrocin or azitromax or Azitromicin or azitromicina or aziwok or azomyne or aztrin or azydrop or azyter or azythromycin or bazyt or cp-62933 or cp62993 or forcin or inedol or infectoazit or isv-401 isv401 kromicin or macrozit or Mezatrin or octavax or ordipha or ribotrex or sumamed or sunamed or tobyl or tromix or trozocina or ultreon or vinzam or xithrone or xz-450 or xz450 or zaret or zarom or zetamax or zeto or zibramax or zifin or zimericina or zistic or zithromax or zithrox or zitinn or zitrim or zitrobifan or zitrocin or zitromax or zmax).tw,kf. (9103)
24 (a56268 or a56268 or abbotic or Abbott-56268 or aeroxina or bactirel or baxin-filmtab or biaxin or biclar or bicrolid or binoklar or bremon or brevil-od or c-clarin or carimycin or celex or cefradine or clacin or clacine or clambiotic or clapharma or clari or claribid or claridar or clarikan or clarimac or claripen or clarith or clarithromycin or clarithromycina or clarithromycine or claritrol or claroma or clormicin or crixan or cylind or cyllind or dicupal or er-36469 or er36469 or gervaken or hecobac or heliclar or helitic or klacid or klacina or klaciped or klaribac or klaricid or klaridex or klaridia or klarin or klerimed or kofron or lagur or lekoklar or macladim or macladin or maclar or macrobiol or makcin or mavid or monoclarium or monozeclar or naxy or soriclar or te-031 or te031 or veclam or winclar or zeclar).tw,kf. (9467)
25 (abomacetin or acneryne or acnesol or akne-mycin or aknederm-ery-gel or aknemycin or anamycin or bonac-gel or c-solve-2 or clinidern or deripil or duraerythromycin or e-mycin or e-base or e-glades or e-mycin or e-solve-2 or emgel or emu-v or emu-ve or emuvin or emycin or eriecu or erimycin-t or eriprodin or eritimix or eritrex or eritrocina or eritromicina or erixyl or ermycin or ermysin or ery-maxin or ery-b or ery-diolan or ery-maxin or ery-tab or eryacne or eryacnen or eryc or eryc-ld or eryc-sprinkles or eryc-125 or eryc-250 or erycen or erycette or erycin or erycinum or eryderm or erydermec or erydermer or eryfluid or erygel or eryhexal or erymax or erymaxin or erymed or erysafe or erystrat or erytab or eryth-mycin or

erythelan or erythmycin or erythomycin or erythra-derm or erythran or erythro-200 or erythro-teva or erythro-statin or erythrogan or erythrogel or erythrogran or erythroguent or erythromid or erythromycin or erythromycine or erythromycinum or erythro
teva or erytop or erytrarco or erytrociclin or etinycine or etrolate or etromycin or ilocap or ilocaps or iloticina or ilotycin or inderm-gel or labocne or latotryd or lederpax or mephamycin or oftalmolosa-cusi-eritromicina or oftamolets or pantodrin or pantomycin or pce or pharyngocin or primacine or r-p mycin or robimycin or romycin or roymicin or rp-mycin or rythocin or sans-acne or sansac or skid-ge-e or staticin or stiemycin or stimycine or t-stat or theramycin).tw,kf. (26599)
26 (coamoxyclav* or coamoxiclav* or co-amoxyclav* or co-amoxiclav* or coamoxyclav* or coamoxiclav* or augmentin*).tw,kf. (14299)
27 or/6-26 (786113)
28 exp placebo effect/ or Placebos/ (39083)
29 (placebo* or no-antibiotic* or no-treatment* or no-intervention*).tw,kf. (253934)
30 28 or 29 (267390)
31 (infan* or toddler* or pre-schooler* or preschooler* or kinder or kinders or kindergarten* or kinder-aged or boy or boys or girl or girls or child or children or childhood or pediatric* or paediatric* or adolescen* or youth or youths or teen or teens or teenage* or school-age* or schoolage* or schoolchild* or schoolgirl* or schoolboy*).af. (4610292)
32 exp *world health organization/ (7181)
33 (WHO or world-health-organization).tw,kf. (2268255)
34 developing countries/ (74943)
35 (austere or (limited adj2 resource*) or (low adj2 resource*) or (transitioning adj econom*) or (third adj world) or LMIC or LMICs or (lami adj countr*) or (transitional adj countr*) or (low adj gdp) or (low adj gnp) or (low adj gross adj domestic) or (low adj gross adj national) or ((emerging or developing or (low adj income) or (middle adj income) or (low adj3 middle) or underdeveloped or under-developed or (less* adj developed) or underserved or under-served or deprived or poor*) and (countr* or nation*1 or econom* or population or world))).tw,kf. (387710)
36 exp africa/ (267131)
37 americas/ or exp caribbean region/ or exp central america/ or latin america/ or mexico/ or exp south america/ (253226)
38 europe/ or exp europe, eastern/ or exp transcaucasia/ (283775)
39 antarctic regions/ or exp atlantic islands/ or exp indian ocean islands/ or exp pacific islands/ (78781)
40 New Guinea/ (2062)
41 asia/ or exp asia, central/ or asia, southeastern/ or borneo/ or cambodia/ or east timor/ or indonesia/ or laos/ or malaysia/ or mekong valley/ or myanmar/ or philippines/ or thailand/ or vietnam/ or asia, western/ or bangladesh/ or bhutan/ or india/ or middle east/ or afghanistan/ or iran/ or iraq/ or jordan/ or lebanon/ or oman/ or saudi arabia/ or syria/ or turkey/ or yemen/ or nepal/ or pakistan/ or sri lanka/ or far east/ or china/ or tibet/ or exp korea/ or mongolia/ (570406)

42 (Afghanistan or Albania or Algeria or Angola or Antigua or Argentina or Armenia* or Aruba or Azerbaijan or Bahrain or Bangladesh or Barbados or Barbuda or Belarus or Byelarus* or Byelorussian or Belorussian or Belorus* or Belize or Benin or Bhutan or Bolivia or Bosnia or Botswana or Brasil or Brazil or Bulgaria or (Burkina adj Fas*) or (Upper adj Volta) or Burma or Burundi or Cambodia or Khmer or Kampuchea or Cameron* or Cameroon* or (Cape adj Verde) or (Cabo adj Verde) or (Central adj African adj Republic) or Chad or Chile or China or Colombia or Comoros or (Comoro adj Island*) or Comores or Mayotte or Congo or Kongo or (Cook adj Island*) or (Costa adj Rica) or (Cote adj D'ivoire) or Croatia or Cuba or Cyprus or Czech* or Djibouti or Dominica or Dominican or (East adj Timor) or (East adj Timur) or Ecuador or Egypt or El-Salvador or (Equatorial adj Guinea) or Eritrea or Estonia or Ethiopia or Fiji or (French adj Somaliland) or Futuna or Gabon or (Gabonese adj Republic)
or Gambia or Gaza or (Georgia* adj Republic) or Ghana or Grenada or Guam or Guatemala or Guinea or Guiana or Guyana or Haiti or Herzeg* or Hercegovina or Honduras or Hungary or India or Indonesia or Iran or Iraq or (Ivory adj Coast) or Jamaica or Jordan or Kazakh* or Kenya or Kiribati or Korea or Kosovo or (Kyrgyz adj Republic) or Kyrgyzstan or Kirghizia or Kirghiz or Kirgizstan or Laos or (Lao* adj2 Democratic adj Republic) or (Lao* adj PDR) or Latvia or Lebanon or Lesotho or Basutoland or Liberia or Libya or Lithuania or Macedonia or Madagascar or (Magalasy adj Republic) or Malawi or Malay* or Sabah or Sarawak or Maldives or Mali or (Marshall adj Island*) or Mauritania or Mauritius or (Agalega adj Island*) or Mexico or Micronesia or Moldov* or Mongolia or Montserrat or Montenegro or Morocco or Ifni or Mozambique or Myanma* or Namibia or Nauru or Nepal or (Netherlands adj Antilles) or (Dutch adj Antilles) or (New adj Guinea) or (New adj Caledonia) or Nicaragua or Niue or Niger or Nigeria or (Northern adj Mariana adj Island*) or Nyasaland or Oman or Pakistan or Palau or Panama or (Papua adj New adj Guinea) or PNG or Palestine or Paraguay or Peru or Philipines or Philippines or Phillipines or Phillippines or Poland or (Puerto adj Rico) or Yemen or Romania or Roumania or Rumania or Russia* or Rwanda or Ruanda or (Saint adj Kitts) or (St adj Kitts) or Nevis or (Saint adj Vincent) or (St adj Vincent) or Grenadines or Samoa* or (Navigator adj Island*) or (Saint adj Lucia) or (St adj Lucia) or (Saint adj Helena) or (St adj Helena) or (Sao adj Tome) or (Saudi adj Arabia) or Senegal or Serbia or Seychelles or (Sierra adj Leone) or Slovenia or Slovak* or (South adj Africa) or (Solomon adj Island*) or Somalia or (Sri adj Lanka) or Ceylon or Sudan or Surinam* or Swaziland or Syria or Tajikistan or Tadzhikistan or Tadjikistan or Tadzhik or Tanzania or Thailand or Tibet or Timor-Leste or Togo or (Togolese adj Republic) or Tokelau or Tonga or Trinidad or Tobago or Tunisia or Turkey or Turkmenistan or Turkmen or Tuval!
u or Uganda or Ukraine or Uruguay or Urundi or USSR or (Soviet adj Union) or "Union of Soviet Socialist Republics" or Uzbekistan or Vanuatu or (New adj Hebrides) or Venezuela or Vietnam or (Viet adj Nam) or (Wallis adj2 Futuna) or (United adj Arab adj Republic) or (West adj Bank) or (West adj Indies) or Yemen or Yugoslavia or Zaire or Zambia or Zimbabwe or Rhodesia).tw,kf. (1271135)

43 (africa or americas or caribbean or (central adj America) or (latin adj America) or (south adj America) or (eastern adj Europe) or Transcaucasia or antarctic or (atlantic adj island*) or (indian adj ocean adj island*) or (pacific adj island*) or polynesia or (central adj asia) or (southeast* adj asia) or (south-east* adj asia) or borneo or mekong or (western adj asia) or (middle adj east) or (far adj east)).tw,kf. (231236)
44 32 or 33 or 34 or 35 or 36 or 37 or 38 or 39 or 40 or 41 or 42 or 43 (4195950)
45 5 and 27 and 30 and 44 and 31 (262)

Table S1: Data extracted from each study

Citation details	• Author<br>• Year<br>• Aim/purpose
Study details	• Publication type<br>• Study type<br>• Data collection period<br>• Age range
Setting	• Clinical setting<br>• Country, region/city<br>• WHO region<br>• World Bank income category<br>• Pneumococcal conjugate vaccine status<br>• *H. influenzae* vaccine status<br>• Role of person who saw participant, both within and outside of the study
Monitoring and supportive care	• Pulse oximetry checked?<br>• Respiratory rate checked?<br>• Clearly defined procedures for escalation of care if required?<br>• Patients observed for how long on first presentation?<br>• For patients managed at home, how frequently is outpatient review?<br>• Documented procedure of education for caregivers of patients managed at home?<br>• For hospitalised patients, how frequently are they reviewed?<br>• Review by a doctor/paediatric specialist for all patients?
Study population	• Number of participants<br>• Age and sex<br>• Inclusion criteria

	• Exclusion criteria<br>• Number of children excluded and reasons for exclusion<br>• Recruitment method<br>• Sampling method<br>• Pre-treatment with antibiotics<br>• Respiratory rate at baseline<br>• Loss to follow-up
Procedure	• Intervention<br>• Control/comparison
Outcomes	All outcomes subdivided into age-specific groups (2-11m, 1-3y, 3-8y, >8y), diagnosis of pneumonia vs bronchiolitis, presence or absence of wheeze, presence or absence of comorbid conditions, including HIV, cardiac disease, malnutrition, and chronic respiratory conditions<br>• Primary study outcome<br>o How was treatment success defined?<br>o How was treatment failure defined?<br>• Secondary study outcome<br>• Other study outcomes<br>• Mortality
Other issues raised	• Acceptability<br>• Feasibility<br>• Practice points
Quality assessment and risk of bias (EPHPP)	• Selection bias<br>• Study design<br>• Confounders<br>• Blinding<br>• Data collection methods<br>• Withdrawal and drop-outs<br>• Overall rating

Table S2: Strength and risk of bias of included studies, according to Effective Public Health Practice Project (EPHPP) ratings

Study	*Selection bias*	*Study design*	*Confounders*	*Blinding*	*Data collection methods*	*Withdrawal and drop-outs*	*Overall*
RCTs comparing antibiotics to placebo in children with WHO-defined fast breathing pneumonia							
Awasthi et al, 2008	Mod	Strong	Strong	Strong	Strong	Strong	Strong
Ginsburg et al, 2019	Mod	Strong	Strong	Strong	Strong	Strong	Strong
Hazir et al, 2011	Mod	Strong	Strong	Strong	Strong	Strong	Strong
Jehan et al, 2020	Mod	Strong	Strong	Strong	Strong	Strong	Strong
Sub-analyses of trials comparing antibiotics to no antibiotics in children with WHO-defined fast breathing pneumonia							
Keitel et al, 2019	Mod	Strong	Strong	Weak	Mod	Strong	Mod
King et al, 2016	Mod	Weak	Weak	Weak	Mod	Mod	Weak
Nkwopara et al, 2019	Mod	Strong	Strong	Strong	Strong	Strong	Strong
RCTs comparing antibiotics to no antibiotics in children with bronchiolitis							
Kabir et al, 2009	Mod	Strong	Strong	Weak	Mod	Strong	Mod
Mazumder et al, 2009	Mod	Strong	Weak	Weak	Weak	Mod	Weak
Pinto et al, 2012	Mod	Strong	Mod	Mod	Mod	Strong	Mod
Rasul et al, 2008	Mod	Strong	Weak	Weak	Mod	Weak	Weak
Tahan et al, 2007	Mod	Strong	Strong	Strong	Strong	Weak	Mod
Non-randomised studies included in this review							
King et al, 2016	Mod	Mod	Mod	Weak	Mod	Mod	Mod
Lassi et al, 2014	N/A	N/A	N/A	N/A	N/A	N/A	N/A

RCTs, randomised controlled trials; WHO, World Health Organization; mod, moderate
